# Are community midwives addressing the inequities in access to skilled birth attendance in Punjab, Pakistan? Gender, class and social exclusion

**DOI:** 10.1186/1472-6963-12-326

**Published:** 2012-09-19

**Authors:** Zubia Mumtaz, Beverley O’Brien, Afshan Bhatti, Gian S Jhangri

**Affiliations:** 1 School of Public Health, University of Alberta, 3-309 Edmonton Clinic Health Academy, 11405 – 87 Ave, Edmonton, AB, T6G 1C9S, Canada; 2 Nursing Faculty, 5–276 Edmonton Clinic Health Academy, Edmonton, AB, Canada; 3 Real Medicine Foundation Pakistan, # 70, Nazimuddin Road, F-7/4, Islamabad, Pakistan; 4 3–319 Edm Clinic Health Academy, Edmonton, AB, Canada

**Keywords:** Pregnancy and childbirth, Pakistan, Maternal health care, Skilled birth attendance, Community-based Midwives, Social exclusion, Inequity, Poverty, Gender, Class

## Abstract

**Background:**

Pakistan is one of the six countries estimated to contribute to over half of all maternal deaths worldwide. To address its high maternal mortality rate, in particular the inequities in access to maternal health care services, the government of Pakistan created a new cadre of community-based midwives (CMW). A key expectation is that the CMWs will improve access to skilled antenatal and intra-partum care for the poor and disadvantaged women. A critical gap in our knowledge is whether this cadre of workers, operating in the private health care context, will meet the expectation to provide care to the poorest and most marginalized women. There is an inherent paradox between the notions of fee-for-service and increasing access to health care for the poorest who, by definition, are unable to pay.

**Methods/Design:**

Data will be collected in three interlinked modules. Module 1 will consist of a population-based survey in the catchment areas of the CMW’s in districts Jhelum and Layyah in Punjab. Proportions of socially excluded women who are served by CMWs and their satisfaction levels with their maternity care provider will be assessed. Module 2 will explore, using an institutional ethnographic approach, the challenges (organizational, social, financial) that CMWs face in providing care to the poor and socially marginalized women. Module 3 will identify the social, financial, geographical and other barriers to uncover the hidden forces and power relations that shape the choices and opportunities of poor and marginalized women in accessing CMW services. An extensive knowledge dissemination plan will facilitate uptake of research findings to inform positive developments in maternal health policy, service design and care delivery in Pakistan.

**Discussion:**

The findings of this study will enhance understanding of the power dynamics of gender and class that may underlie poor women’s marginalization from health care systems, including community midwifery care. One key outcome will be an increased sensitization of the special needs of socially excluded women, an otherwise invisible group. Another expectation is that the poor, socially excluded women will be targeted for provision of maternity care. The research will support the achievement of the 5^th^ Millennium Development Goal in Pakistan.

## Background

Complications of pregnancy and childbirth remain the leading cause of death and disability for childbearing women in Pakistan. With a measured maternal mortality rate of 297/100,000 live births, Pakistan is one of the six countries^1^ estimated to contribute to over half of all maternal deaths worldwide [1]. The lifetime risk of maternal death is one in 89 for Pakistani women, compared to 1 in 17,400 in Sweden [2].

**Figure 1:**
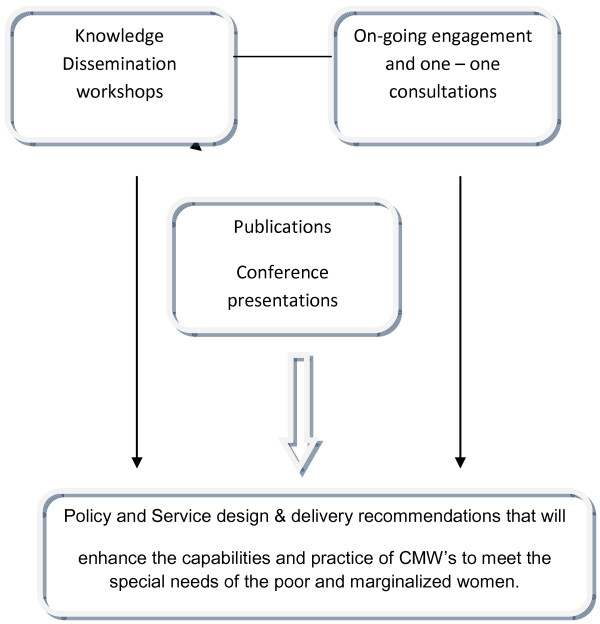
Knowledge dissemination and desired policy/practice change.

Pakistan is also characterised by vast inequalities in maternal health status and access to health care services. For example, 77% of women in the wealthiest quintile report care by skilled birth attendants, compared to 16% of women in the poorest quintile. Women with higher education are over three times more likely to be attended by skilled birth personnel than are women with no formal education (86% vs. 27%) [1].

To address these inequities, the government of Pakistan created a new cadre of community-based midwives (CMW) [3]. Launched in 2006, the program trains and equips locally resident women to provide basic obstetric care. Although these midwives operate in a fee-for-service context, a key expectation is that they will improve access to professional antenatal and intra-partum care for poor and disadvantaged women.

A critical gap in our knowledge, however, is whether this cadre of workers, operating in the private health care context, will meet the expectation to provide care to the poorest and most marginalized women. There is an inherent paradox between the notions of fee-for-service and increasing access to health care for the poorest who, by definition, are unable to pay. In particular, we know very little about how class and caste membership (in addition to poverty) may influence women’s access to CMW services. There is a paucity of such research in Pakistan, but a growing body of evidence from South Asia and elsewhere shows that the large disparities in access to maternal health care between the rich and poor are not just the result of economic poverty [4,5]. Economic poverty is relational and embedded within power hierarchies influenced by both class and gender [6,7]. In Pakistan, perceptions of social identity are also related to caste membership [8]. Powerful groups construct and draw upon societal beliefs, norms and values to disparage, make invisible and demean certain groups of people and, thereby justify denying them full rights of participation in economic, social and political life [9]. Caste membership and gender are inextricably linked with economic poverty [10]. The concept of social exclusion may therefore be a more useful framework to draw upon to gain an in-depth understanding of potential disparities that may exist in access to CMW services. Originally developed in French political discourse [11], the social exclusion framework is well developed in understanding the relational aspects of deprivation, how systematic societal structures that sharply define hierarchical group boundaries serve the interests of a privileged group and marginalize others [12-17]. It is also a useful framework to generate a greater understanding of how women in Pakistan, as a gendered group, may be socially excluded. By focusing attention on social relations, of both class and gender, the social exclusion framework will enable us to understand disparities in poor women’s access to CMW services within a single analytic framework.

It is important to explore whether the CMW’s will provide care to marginalized/socially excluded women because health systems, of which the CMW’s are a part of, are not just mechanical structures; they “are also purveyors of a wider set of societal norms and values” (pg. 143) [18]. According to Freedman, health systems are “core social institutions, culturally embedded, politically contingent, and part of the very fabric of social and civic life” (pg. 22) [19]. More importantly, they are a common interface between individuals and the power structures that shape their broader society [18]. Neglect, abuse and exclusion within health care systems, common in Pakistan, may thus essentially be a reflection of the experience of being poor and socially marginalized in that society [20,21].

It is necessary, but not sufficient, for health systems to ensure that good quality maternity services are widely available; utilization of these services also involves the dynamics of users’ decision-making. A large body of literature addressing the three-delays-model [22] of maternal health care utilization in developing countries has tended to focus on the individual characteristics of women, their families and decision-making processes. This individualistic approach fails to consider how health system characteristics *per se* may shape the decision-making process, including the primary decision of whether or not to seek care at all. Poor quality, differential and inequitable services negatively impacts women’s perceptions and use of physically available maternal health care services [23].

There is, therefore, an urgent need to critically analyse whether the CMW program is overcoming these structural barriers and providing care to the poor, less educated and socially excluded/marginalized women at this stage of the program. An understanding of these factors will provide policymakers and the CMW program managers with an enhanced understanding of underlying structures and processes that perpetuate disparities in access to maternal health care and provide much needed insight into addressing what can appear to be insurmountable, deep seated inequalities and problems.

## Research questions

Drawing on work by Freedman *et al.*[20], the proposed research aims to explore if the CMWs are achieving the government objective of improving access to the full scope of skilled maternity care (antenatal, intra-partum and postnatal care) for the poor and disadvantaged women in Pakistan. Specifically the study will investigate

(1) Are the CMWs providing maternity services to poor and/or socially excluded women? Are they more likely to provide care to the poor and marginalized women or the less poor or richer women?

(2) What challenges (organizational, social, financial) do the CMWs face in providing care to the poor and socially marginalized women? Can a young unmarried CMW (Over 60% of the trained CMW are young, unmarried women) discuss a woman’s issues or complications during pregnancy and childbirth with the husband who is - in the local context of sharp *biradari*^2^ boundaries - a ‘*gaer mard’*^3^? How do they balance their income generation requirements with the fact that the poor, by definition will be unable to pay?

(3) What social, financial and other barriers do poor and socially excluded women face in accessing CMW services? Do the financial costs of seeking care from a CMW preclude poor women seeking such care Do they believe that CMW’s treat them differentially because of caste*,* economic and other social hierarchies?

The key variables in the proposed research will include context-specific, measurable indicators of social exclusion, birth attendance by the CMW and indicators of quality and satisfaction with the care provided by the CMW. The development of social exclusion indicators will be informed by the work of Hooper,and Hamid [24], Popay *et al.*[25], Carraro [26], Mohmand and Gazdar [27] and an on-going University of Alberta, Canadian Institutes of Health Research-funded study that is in the process of developing a locally-informed, contextually-relevant conceptual framework to understand the construction and experience of key axes of social exclusion relating to gender, class, and caste in Punjab, Pakistan [28]. The Principal Investigator (ZM) of the present proposal is the PI of the latter study. Based on our preliminary data and the literature we foresee the social exclusion index containing indicators of caste, landholding, husband’s education and occupation, woman’s education and involvement in income generating work, home ownership and children’s schooling. The indicators for birth attendance will include whether the CMW personally attended the labour and birth in any setting (home, hospital, health centre). The indicators of quality of care will include women’s first trimester access to CMW; a minimum of four comprehensive antenatal visits; tetanus toxoid vaccination and iron and folic acid supplementation. Indicators for quality of care during birth will include monitoring progress of active labour and maternal and fetal well-being. Indicators of satisfaction with CMW care include women’s satisfaction with the respect they are accorded, their sense of dignity, equity and emotional support, the information they are provided regarding their pregnancies, and the usefulness of the procedures they undergo. Since a key objective of CMW deployment was to reduce maternal deaths by identifying, stabilizing and referring complicated births, a set of indicators will measure maternal perception of CMW’s recognition of complications and subsequent care provided.

Several outcomes of the proposed research are anticipated. First, the research will provide policy recommendations for (1) Inputs into the CMW training curriculum and in service training (2) Improvement in design and delivery of CMW services and (2) changes in CMW professional practice to increase the CMW’s awareness and sensitivity to the unique needs of the poor and marginalized women, whilst ensuring CMW’s are equitably compensated. The research will also generate potentially transformative theoretical advances in understanding power dynamics of gender and class that may underlie poor women’s marginalization from health care systems in Pakistan.

## Methods/Design

A mixed methods approach using both quantitative and qualitative methods will be used. Data will be collected in three overlapping modules over a three year period.

1. Module 1 will address research question 1 and consist of a population-based survey in the catchment areas of the CMW’s.

2. Modules 2 and 3 will answer research question 2 and 3 using qualitative research methods with an Institutional Ethnographic approach. See Figure 1

### Module 1

This module will use a population based cross-sectional survey to

(1) Assess what proportions of the socially excluded women are served by different cadres of service providers. Are they more likely to access services of the CMWs than other skilled providers (doctors, nurses) or are they more likely to be attended by *dai’s* and other untrained personnel?

(2) Assess poor and socially excluded women’s levels of satisfaction with their maternal health care provider. Are these women more or less satisfied with the services of a CMW compared to other providers (doctors, *dai*, family members)?

(3) Explore differentials in birth outcomes/complications by type of provider? Do women attended by CMWs have birth outcomes/complications similar to women attended by other trained providers? Does she make appropriate referrals? Do the referrals vary by women’s social exclusion status?

#### Study population

Since the CMW training program is on-going, two districts in which large numbers of CMWs have been deployed will be selected. To ensure representativeness, one district will be well-developed (Jhelum) and one less-developed (Layyah). The target population consists of women aged 15–49, who have given birth in the three years prior to the survey and live in rural areas of these CMW-served districts. The three-year limit is used because the first batch of CMW’s graduated in 2008.

#### Sampling and sample size

A cross-sectional, clustered and stratified survey will be conducted. A survey is a method of choice for module 1 for it will enable us to quantitatively measure what proportion of pregnant women in Districts Jhelum and Layyah are cared for by the CMWs compared to other providers, in particular *dais* and other untrained and unskilled providers. Sample size was calculated using the statistical package PASS 2000 [28] program and is based on numbers reported in the 2006–07 Pakistan Demographic Health survey (PDHS) [1]. Since social exclusion is a complex notion not yet measured in surveys in Pakistan, we took socio-economic status index (SES) as a proxy indicator of social exclusion for the purposes of calculating sample size. The PDHS data show that the probability of skilled attendance at birth ranges from 0.25 in lower half of the SES index to 0.5 in upper half of the index, which translates into an odds ratio of 3.0. To be conservative, we chose an OR of 1.8. In order to detect this difference with 80% power and 0.05 significance level, based on a logistic regression model of a binary outcome variable (skilled attendance at birth) on a binary independent variable (SES), a minimum sample size of 880 women will be necessary. This sample size takes into account a multiple correlation between SES and other predictor variables of 0.6 and a design factor of 1.3.

To interview 880 women, we estimate a total of 40 primary sampling units will be required. A primary sampling unit in this survey has been identified as a village. The sampling frame will be drawn up using a three stage sample design. District Jhelum is subdivided into four tehsils and 53 union councils. District Layyah is subdivided into three tehsils and 90 union councils. In the first stage, two out of four tehsils in each district will be randomly selected (total 4 tehsils). In the second stage, five union councils will be randomly selected in each of the selected tehsils (total 20 unions councils). In the third stage, two villages will be randomly selected from each of the selected union councils (total 40 villages). An inclusion criteria is that the village randomly selected is served by a CMW. If a selected village is not served by a CMW, it will be excluded and we will select the next village until we reach a CMW-served village. Within each selected village, 22 women who gave birth in the three years prior to the survey will be interviewed [29]. In order to ensure that sufficient numbers of the poor and socially excluded women are represented in the sample, we will ensure that at least 11 of the 22 women interviewed in each village are landless and belong to the lower status castes. The first house to be contacted will be the house next to the first mosque we see in the village. Every second house will be visited. To find 22 women aged 15–49 years who have given birth in the past three years, we need to *contact* at least 124 households per village. In some of our selected villages, we may not be able to find 22 targeted women and so will continue contacting more households in the nearest neighbouring village until we hit our target. The number of households we need to contact is based on a demographic assumption that 70% of households will have a woman aged 15–49, 30% of whom gave birth in the last three years [1] and a participation refusal rate of 15% (all conservative estimates). If there is a woman aged 15–49 who gave birth in the past three years present in the house, the household will be selected and the woman will be interviewed. Data will be collected using a pre-tested questionnaire that will include a set of questions aimed at populating the indicators described in section 2 above. This will include, but not limited to identifying the socially excluded (by asset ownership, including land-ownership, caste, education), attendance at birth (type of attendant, place of delivery), the financial costs, including hidden costs, associated with the birth and satisfaction with the care provided. We will continue visiting homes and interviewing women until at least 11 poor and socially excluded women are interviewed. This might mean that more than 22 women may be interviewed in each village. The women will be interviewed face to face in a private setting. If the woman cannot be interviewed at the time of contact, a mutually agreed upon time and place will be scheduled for another visit and interview. Women will be interviewed by female enumerators. If the women are unable to answer questions regarding landownership or other questions, the house will be revisited when the husband is available. The husbands will be interviewed by male interviewers. It is anticipated that most interviews will last approximately 45 to 60 minutes.

##### Data analysis

Data will be analyzed using Stata 11.0 [30]. We will first develop an index of social exclusion using principal component analysis. Caste, land ownership, home ownership, husband’s education and occupation are some of the variables that will be used to develop the social exclusion index. The index will be scaled and ranked into quintiles. The top 20% will consist of the rich and ‘socially included’, while the bottom 20% will be the poorest and socially excluded. Univariate and bivariate analyses will be done to assess the size and distribution of the socially excluded groups, the type of attendant at birth and relationships between social exclusion status and attendance by CMW and satisfaction with care provided. Logistic regression models will then be developed to estimate the odds of attendance by CMW (and other skilled attendants) by social exclusion status, controlling for potential independent predictors and confounders. Are the more marginalised women more or less likely to use a CMW compared to a ‘more socially included’ woman? How satisfied are the socially excluded women with the care provided by the CMW’s compared to the more ‘socially included’ women? Is there any variability in birth outcomes between the two groups of women under the care of a CMW?

### Module 2

This module will provide an empirically grounded exploration of the challenges (organizational, social, financial) that CMWs may face in providing care to the poor and socially marginalized women. We will use qualitative research methods, specifically an institutional ethnography as these methods will enable us to piece together a picture of how the conduct of people’s lives in any institution is co-ordinated in relation to ruling ideas and practices [31]. By starting with individual accounts, such as a CMW talking about type of care she provided to a woman in childbirth, observing patient-provider interactions and the process of care during childbirth, an understanding will be developed of how these acts are embedded in interconnected ruling systems (where does the CMW refer the patient when needed, how these decisions depend on patients social and financial status), and mapping out these systems (where is the patient taken, who accompanies her, the financial and social costs involved) an institutional ethnography, by producing a detailed account of the events, will identify the challenges the CMW may face in providing care to the poor women. These methods will also enable us to develop a ‘thick description’ of the subtleties of power and resource distribution operating in this context and how CMWs negotiate around them.

#### Study population and sample size

Sample size in qualitative research is difficult to ascertain and specify in advance, particularly in ethnographic designs where multiple sources of data are appropriate in gaining important insights. However, with maternity care policy as institution (i.e. area wherein power relationships that shape service delivery are embedded) and considering that 12 interviews are usually enough to reach data saturation [32], we estimate we will need to interview 20 CMW’s, 10 in each district (Jhelum and Layyah). The CMW’s will be randomly selected from lists of CMW’s trained and deployed maintained by the National Maternal Newborn and Child Health Program, Punjab. Ten managers and policymakers of the CMW program will also be interviewed. These individuals will be identified from the MNCH organogram. We also anticipate that the CMW’s will identify other leaders who exercise informal power and influence. If such individuals are identified, they too will be invited for an interview. An interview guide will be developed, but will be refined throughout the course of data collection so that we can explore in-depth critical concepts revealed in the initial interviews. The respondents will be free to guide the discussion in ways they deem fit. In addition, 20 moment-in-time patient provider interactions in routine antenatal and postnatal visits will be observed. Five women delivering under the care of a CMW will also be observed for the duration of childbirth and three days postpartum. The points of observation will include, but are not limited to list of services the CMW is supposed to provide as part of the CMW care package (monitoring labour progress, monitoring maternal and fetal wellbeing, infection control techniques), to the CMW’s language and behaviour towards the patient and her family, time spent with the patient and whether her concerns are addressed. Any complications or adverse outcome will be explored in depth, which will include interviewing the women and her family.

### Module 3

This module aims to identify the social, financial, geographical and other barriers, in particular to uncover the hidden forces and power relations that shape the choices and opportunities of poor and marginalized women in accessing CMW services. Using the Institutional Ethnographic approach, which is consistent with our intent to uncover power relationships that influence the maternity care of the most marginalized women, in-depth interviews and focus group discussions will be held in two randomly selected villages, one in each district Jhelum and Layyah. In depth and group interviews are useful methods for not only exploring people’s understandings, interpretations and experiences of how gender, caste and class impact upon their ability to access CMW’s, but also of the language they use in constructing the discourse related to these experiences [33]. Other sources of institutional power may that shape the maternity experience of women e.g., husbands, mothers-in-law, local political leaders.

#### Study population and sample size

Respondents will include (1) women of reproductive age (15–49 years) that have given birth in the last 3 years; (2) husbands of women who gave birth in the past three years (3) ten older women (over the age of 50) since they are often the primary decision-makers around maternal health issues [34]. All respondents will belong to socially excluded groups as defined in Module 1 (lower status caste, landless). We estimate we will need to conduct about 20 in-depth interviews with women, 20 with men and 10 with older women. We will also conduct 8 focus group discussions, with 6–10 participants in each, separately for women and men [35]. Representation of all *castes and* socio-economic groups will be ensured in these group discussions.

Respondents will be recruited using snowballing techniques. The snowball sampling approach, which falls under the broader category of purposeful sampling, involves identifying a person or “case of interest” and then asking that person to recommend other potential participants who would also be appropriate and asking subsequent participants to recommend more potential participants [36]. The initial respondents will be identified by the local community health worker, commonly known as the Lady Health Worker (LHW) in the villages that we know from module 1 are served by a CMW. They maintain household registers, which includes data on all births in their covered areas and as local residents, are privy to information about villagers’ caste and landownership. Great care and sensitivity will be exercised in identifying these groups as membership of a lower status caste and poverty are stigmatising. The interviews and focus group discussions will be conducted using pre-tested guides. We may also examine printed materials that are available locally and relate to maternity care e.g., advice provided by professional or older women.

##### Data analysis

Data in Modules 2 and 3 will be collected using participant and non participant observation, audio recorded individual and group discussions, field notes and available data including community demographics. All formal interviews will require participant consent following a clear presentation of their rights as well as risks/benefits of study participation. Since the data will be collected in *Punjabi and Seraiki*, the local dialect, data will be translated and transcribed by native Punjabi and Seraiki speakers. The PI (ZM) and research manager (AB) (who understands and speaks Punjabi and Seraiki respectively) will independently (back-translate) all interview transcripts based on audio-tapes to verify the translation and focus on conceptual equivalence. Any discrepancy with original translation will be discussed until consensus is reached with respect to the meaning. A database of the transcribed interviews, focus groups, and observation notes will be created in Atlas-ti, a qualitative data analysis software program [37]. Using a social constructivist, interpretative approach [38], data will be coded and broad themes identified. Initial coding will be guided by the stated research objectives and later by additional concepts as they emerge. Data analysis will be an on-going and iterative process through all phases of data collection, as early identification will allow a fuller probing of unanticipated concepts and variables in upcoming formal or informal interviews and focus group discussions [39]. An audit trail will be maintained throughout the research process to insure that other researchers are able to follow the decision trail. This will be achieved through personal memos and journaling throughout the data collection and analysis [40]. Interpretive accuracy will be assessed by triangulation of findings across the four phases, peer debriefing within the research team and other colleagues and respondent validation [33]. Researcher bias will be reduced through researcher training, peer discussions and respondent validation.

##### Data security

Maintaining privacy and confidentiality of all the study participants is an important ethical issue. This will be addressed by only collecting the necessary information [41]. All data and any confidential information, such as the names and other identifying information that will known to the researchers, will be put into password protected documents on the research project’s computer. Each participant in modules 2 and 3 will be assigned a code and names and other identifying information will never used. Only the researchers and project manager will have access to the list of participants’ names and associated codes. The hardcopies of the survey questionnaires, the qualitative data transcripts and consent forms will be kept in a locked cabinet in the research project office in Islamabad. On completion of the research, all confidential information will be burned onto an external memory storage device and stored in a locked cabinet in at the University of Alberta for five years according to the University of Alberta’s Human Research Ethics Board guidelines [42].

##### Ethical considerations

Ethics approval has been obtained from the University of Alberta Health Research Ethics Board and the Pakistan Medical Research Council National Bioethics Board. Confidentiality, voluntary, informed participation and safety of participants will be given priority during the research process. Gender, class and caste are sensitive issues. Gender is a contentious issue in Pakistan; men in particular are hostile to the notion that gender inequities exist. The caste system is not so contentious, but it is not something people talk about openly, especially those who belong to the lower status castes. Whilst the higher caste people proudly display their caste in their names, poverty and low status caste are stigmatizing. We will therefore not direct ask questions related to these subjects; they will however form the theme upon which our questions will be based. The PI (ZM) and research manager (AB) have extensive experience handling such delicate questions and can steer conversations to get maximum information without hurting sentiments. Written consent will be obtained from the CMW and policymaker and program managers. However, oral consent will be obtained from the poor, socially women and men who will be interviewed in module 3. It is important to limit the consent process to oral consent only because the research will be conducted in a rural area with low levels of education. Requests for signatures in this context are viewed with a high degree of suspicion for it indicates a legal document is being signed, over which they perceive they will have little control. Data security of both qualitative and quantitative data will be ensured as described in section 2 above.

## Discussion

The ultimate goal of this research project is to improve maternal health service delivery by CMWs by identifying and addressing the critical barriers that marginalized populations may face in accessing their services. While policymakers are clearly open to improving services, there is often an inadequate understanding of the deeply rooted multi-dimensional inequities embedded within societal structures. The insights to be gained from this study are clearly needed.

We expect the findings of the proposed research will lead to improvements in the delivery of maternity care for poor and socially excluded women. One key outcome we expect is an increased sensitization regarding the existence and special needs of poor marginalized women, primarily through their training. This is an important outcome because this group of women are invisible [43]. Due to the sensitive and contentious nature of discussions involving gender, caste and related social distinctions, a key element of engagement with various stakeholders will be to encourage open and thoughtful engagement with realities that are typically uncomfortable to address.

Another expectation is that the poor and socially excluded women will be targeted for provision of maternity care. This of course first requires identifying the socially excluded women in a way that captures the complexity of the exclusion (the caste-based exclusion for example), but is not at the same time stigmatizing. A current ongoing project of this research team will contribute to this identification [28]. Some ways of targeting include compensating CMWs for providing care to poor women or providing the poor with vouchers that can be used for CMW care. Another possibility is developing a compensation system that would reward the CMWs for providing care to socially excluded women and reducing the attractions and advantages of providing care to wealthier women. Other areas that could be addressed include addressing access issues for the women themselves (geographic, social, family resistance etc., abusive care givers). Strategies to reduce access issues that might be considered include compensating women and their families for utilizing CMW care.

## Knowledge translation

Stakeholders will be engaged to critically reflect on the research findings and to assess their applicability to CMW policy development, service design and delivery throughout the three-year research program. The key stakeholders of this research are the (1) Maternal and Neonatal Child Health program policymakers, managers, CMW curriculum developers and trainers, at both the federal and provincial levels (2) Community Midwives (3) Community members (4)Civil society groups that provide maternal health care such as the Family Planning Association of Pakistan, Marie Stopes and others (5) International organizations such as the UNFPA, the International Council of Midwives (the group that developed the current CMW training curriculum), UNICEF, and WHO.

The knowledge dissemination activities include

1. Knowledge dissemination workshops: A total of two knowledge dissemination workshops will be conducted.

2. On-going engagement and one-one consultations with community stakeholders and stakeholders in various professional and governmental groups will occur throughout the research program.

3. Research findings will be shared with peer researchers and academics at national and international conferences.

4. Input will be offered to curriculum and in-service training for CMWs.

5. Publications will include short briefing papers, contributions to professional newsletters, press-releases and publications in international peer-reviewed journals.

After this three-year project has concluded, and contingent upon interest among local authorities, the research team is willing to provide advice and assist in the development of pilot interventions based on the research findings. A follow-up research funding proposal will include monitoring subsequent changes to policy and practice.

## Endnotes

^1^ The six countries are India, Nigeria, Pakistan, Afghanistan, Ethiopia, and the Democratic Republic of Congo

^2^ A *biradari* is a group of households related by blood, essentially the large extended family. It is however, also the basic social, political and economic unit in Pakistan [27,44].

^3^ A ‘*gaer mard’* is a man who does not belong to the woman’s *biradari*. In a context of gendered norms of *purdah* (seclusion), women, especially young unmarried women, are not expected to socially interact with men who do not belong to their *biradari*.

## Abbreviations

CMWs: Community-based Midwives.

## Competing interests

The authors declare that they have no competing interests.

## Authors’ contributions

ZM, BB, AB conceptualized the study. ZM and BB prepared the manuscript. GJ provided statistical input. All authors approved the final submission.

## Authors’ information

ZM (MBBS, MPH, PhD), Alberta Heritage Foundation for Medical Research Population Health Investigator and Assistant Professor, School of Public Health, University of Alberta. She specializes in gender and reproductive health issues with a particular focus on women’s access to reproductive health services and inequities in reproductive health policy, design and delivery of services.

BB (RN, PhD), Professor, Faculty of Nursing, University of Alberta is a registered midwife and nurse whose research has focused on maternity care.

GJ Associate Professor, School of Public Health, University of Alberta, is an applied biostatistician.

AB (Msc Anthropology and MBA (Germany) is the Research Manager and Anthropologist coordinating University of Alberta’s research projects in Pakistan.

## Pre-publication history

The pre-publication history for this paper can be accessed here:

http://www.biomedcentral.com/1472-6963/12/326/prepub
